# *Herba houttuyniae* Extract Benefits Hyperlipidemic Mice via Activation of the AMPK/PGC-1α/Nrf2 Cascade

**DOI:** 10.3390/nu12010164

**Published:** 2020-01-07

**Authors:** Ke Cao, Weiqiang Lv, Xuyun Liu, Yingying Fan, Kexin Wang, Zhihui Feng, Jianshu Liu, Weijin Zang, Lianxi Xing, Jiankang Liu

**Affiliations:** 1 Center for Mitochondrial Biology and Medicine, The Key Laboratory of Biomedical Information Engineering of Ministry of Education, School of Life Science and Technology, Xi’an Jiaotong University, Xi’an 710049, Shaanxi, China; caoke@stu.xjtu.edu.cn (K.C.); wqlv_xjtu@163.com (W.L.); liuxuyun2015@126.com (X.L.); peacock5@163.com (Y.F.); KexinWangxjtu@126.com (K.W.); zhfeng@mail.xjtu.edu.cn (Z.F.); 2 Frontier Institute of Science and Technology, Xi’an Jiaotong University, Xi’an 710049, Shaanxi, China; 3 Shaanxi Translational Center for Functional Foods, Xi’an 710065, Shaanxi, China; JianshuLiu111@126.com (J.L.); lxxing@nwu.edu.cn (L.X.); 4 Department of Pharmacology, Xi’an Jiaotong University Health Science Center, Xi’an 710061, Shaanxi, China; zwj@mail.xjtu.edu.cn

**Keywords:** *Herba houttuyniae*, hyperlipidemia, AMP-activated protein kinase, mitochondrial biogenesis, oxidative stress

## Abstract

Hyperlipidemia is associated with metabolic disorders, but the detailed mechanisms and related interventions remain largely unclear. As a functional food in Asian diets, *Herba houttuyniae* has been reported to have beneficial effects on health. The present research was to investigate the protective effects of *Herba houttuyniae* aqueous extract (HAE) on hyperlipidemia-induced liver and heart impairments and its potential mechanisms. Male C57BL/6J mice were administered with 200 or 400 mg/kg/day HAE for 9 days, followed by intraperitoneal injection with 0.5 g/kg poloxamer 407 to induce acute hyperlipidemia. HAE treatment significantly attenuated excessive serum lipids and tissue damage markers, prevented hepatic lipid deposition, improved cardiac remodeling, and ameliorated hepatic and cardiac oxidative stress induced by hyperlipidemia. More importantly, NF-E2 related factor (Nrf2)-mediated antioxidant and peroxisome proliferator-activated receptor gamma coactivator-1 alpha (PGC-1α)-mediated mitochondrial biogenesis pathways as well as mitochondrial complex activities were downregulated in the hyperlipidemic mouse livers and hearts, which may be attributable to the loss of adenosine monophosphate (AMP)-activated protein kinase (AMPK) activity: all of these changes were reversed by HAE supplementation. Our findings link the AMPK/PGC-1α/Nrf2 cascade to hyperlipidemia-induced liver and heart impairments and demonstrate the protective effect of HAE as an AMPK activator in the prevention of hyperlipidemia-related diseases.

## 1. Introduction

Metabolic syndrome (MS) has become one of the major diseases affecting human health. Hyperlipidemia, hyperglycemia, and hypertension are considered to be major symptoms and central risk factors of MS [1,2,3]. Hyperlipidemia is characterized by excessive lipids (mainly triglycerides, cholesterol, and free fatty acids) in the bloodstream [4]. Previous studies have suggested that hyperlipidemia, which is closely related to the occurrence of metabolic abnormalities such as fatty liver disease [5], diabetes [6], and cardiovascular diseases [2,4], causes damage to a variety of tissues. 

Although the detailed mechanisms by which hyperlipidemia contributes to metabolic diseases remain unclear, evidence has suggested that, due to hyperlipidemia, excessive triglycerides (TG) and cholesterol (TC) are deposited in blood vessels and circulate to other organs, possibly leading to lipid metabolic disorders and tissue impairment and eventually increasing the risk of MS [7]. Apart from excessive lipid deposition, oxidative stress [8,9] and mitochondrial dysfunction [10,11] have also been demonstrated to be closely linked to hyperlipidemia-related tissue impairment. A clinical study revealed that mitochondrial DNA (mtDNA) copy numbers are significantly reduced in patients with hyperlipidemia [11]. Moreover, investigations have shown that excessive free fatty acids (FFAs) can amplify reactive oxygen species (ROS) generation, leading to increased oxidative stress and decreased mitochondrial oxidative capacity, which eventually causes morphological and functional changes in tissues [8,12]. 

Adenosine monophosphate (AMP)-activated protein kinase (AMPK) has become an attractive target for many diseases due to its role in regulating energy metabolism [13,14,15]. AMPK deficiency has been reported to induce cardiac contractile dysfunction and dilated cardiomyopathy [16], and the loss of AMPK activity can increase hepatic steatosis and promote fatty liver disease [15,17]. In addition to modulating lipogenesis and peroxisome proliferator-activated receptor gamma coactivator-1 alpha (PGC-1α)-mediated mitochondrial biogenesis [15,18], both recent studies [19,20] and our studies [21,22] have revealed that AMPK can also regulate oxidative stress through NF-E2 related factor (Nrf2)-mediated phase II antioxidant enzymes. Evidence has also suggested a correlation between hyperlipidemia and the AMPK pathway in the vascular system [23,24]. We observed obese mice or rats with hyperlipidemia exhibit decreased AMPK activity accompanied by mitochondrial dysfunction and oxidative stress in various organs [22,25,26]. However, most previous studies have been based on a complex and indirect model of hyperlipidemia, and the mechanisms of hyperlipidemia-induced tissue impairment remain unclear, especially in the early stage of the disease. Thus, understanding of the effects of AMPK-regulated mitochondrial biogenesis and oxidative stress in a direct model of hyperlipidemia is limited and requires further elucidation. Here, we used poloxamer 407, a nonionic surfactant that has been reported to cause hyperlipidemia in animals [27], to investigate the regulatory role of AMPK and its related downstream pathways in the liver and heart of a mouse model of acute hyperlipidemia. 

*Houttuynia cordata Thunb*, also known as *Herba houttuyniae* or *Houttuyniae herba*, is not only a widely distributed medicinal plant but also a functional food with important biological actions, mainly including anti-inflammatory [28], antiviral [29], antioxidant [30], and antitumor [31] effects. Recently, a few studies have shown that *Herba houttuyniae* may also play a protective role in diabetes because of its anti-inflammatory activity [30,32]. However, the beneficial effects of *Herba houttuyniae* on hyperlipidemia-associated abnormalities still unclear. In the current research, we explored the detailed mechanism of acute hyperlipidemia-induced metabolic disorders and tissue impairment and the potential protective effects of *Herba houttuyniae* aqueous extract (HAE), with a focus on the AMPK/PGC-1α/Nrf2 cascade.

## 2. Materials and Methods 

### 2.1. Chemicals

Antibodies against β-actin, glyceraldehyde-3-phosphate dehydrogenase (GAPDH, #5174), fatty acid synthase (FAS, #3180), acetyl-coenzyme A carboxylase 1 (ACC1, #4190), p-AMPK (#2535), and AMPK (#2532) were acquired from Cell Signaling Technology (Danvers, MA, USA). Antibodies against NAD(P)H/quinone oxidoreductase (NQO1, #sc-376023), heme oxygenase-1 (HO-1, #sc-390991), NF-E2 related factor (Nrf2, #sc-13032), carnitine palmitoyltransferase-1L (CPT1L, #sc -377294), manganese-containing superoxide dismutase (MnSOD, #sc-137254), mitofusin-1 (Mfn1, #sc-50330), and mitofusin-2 (Mfn2, #sc-50331) were acquired from Santa Cruz Biotechnology (Santa Cruz, CA, USA). Antibodies against complexes I (39 kDa, #459130), II (30 kDa, #459230), III (51 kDa, #459140), IV (40 kDa, #459600), and V (55 kDa, #459240) were acquired from Invitrogen (Carlsbad, CA, USA). Antibodies against optic atrophy 1 (OPA1, #612607) and dynamin-related protein 1 (Drp1, #611113) were acquired from BD (Franklin Lakes, NJ, USA). An antibody against peroxisome proliferator-activated receptor gamma coactivator-1 alpha (PGC-1α, #TA319007) was acquired from OriGene Technology (Rockville, MD, USA). Poloxamer 407 (#P2164030) was acquired from Sigma Aldrich, St. Louis, USA. *Herba houttuyniae* aqueous extract (HAE, catalog, BLT20170412) was acquired from Xi’an Brilliant Chem Co., Ltd. (Xi’an, Shaanxi, China). Briefly, the aerial part of fresh *Herba houttuyniae* was washed and dried in a constant temperature-drying box. After being crushed into 10-mm fragments, distilled water was added according to the liquid-to-material ratio of 20:1, followed by reflux extraction at 90 °C for 2 h. The filtrates were dried under vacuum and passed through an 80-mesh sieve to obtain the HAE powder. The active chemical constituents in HAE mainly include flavonoids, alkaloids, organic acids, polyphenols, and polysaccharides [28,29,30].

### 2.2. Animals and Treatment

Male C57BL/6J mice at the age of eight weeks old were acquired from Vital River Laboratory Animal Technology Co., Ltd (Beijing, China). The mice were divided into four groups at random (*n* = 8 in each group): the control group (Con), the poloxamer 407 (P407)-treated group (P407), the P407-treated group with a daily oral gavage of a low-dose HAE (200 mg/kg/day) (P407 + HAE), and a high-dose HAE (400 mg/kg/day) group. An HAE gavage was administered from day 1 to day 9. On day 10, the mice were intraperitoneally injected with P407 (0.5 g/kg) to cause acute hyperlipidemia for exactly 24 h before sacrifice, and the mice in the Con group were injected with saline. All the procedures were performed in accordance with the National Institutes of Health guide for the care and use of laboratory animals (NIH Publications No. 8023, revised 1978) and approved by the Animal Care and Use Committee of the School of Life Science and Technology, Xi’an Jiaotong University (2019–0012). 

### 2.3. Biochemical Analysis

Tissue homogenate and serum samples were obtained according to the previous method [23]. Reduced glutathione (GSH), oxydized glutathione (GSSG), triglyceride (TG), and total cholesterol (TC) contents as well as glutathione S-transferase (GST), glutathione peroxidase (GPX), γ-glutamylcysteine synthetase (γ-GCS), and total superoxide dismutase (SOD) activities were analyzed by using detection kits (Jiancheng, Nanjing, China). Adenosine triphosphate (ATP) level was determined by using a bioluminescent kit (Sigma Aldrich, St. Louis, MO, USA) [33]. The serum contents of free fatty acids (FFAs), malondialdehyde (MDA), and cholesterol low-density lipoprotein (c-LDL) and the activity of lactate dehydrogenase (LDH) and alanine aminotransferase (ALT) were determined by using detection kits (RD Systems, Shanghai, China).

### 2.4. Histological Analysis

Liver and heart tissues were placed in 4% paraformaldehyde and sliced 3–4 μm thick. Then the tissues were stained with hematoxylin and eosin (H&E) and visualized by an Olympus BX71 microscope. 

### 2.5. Mitochondrial Complex Activity Analysis

Fresh liver and heart mitochondria were isolated using a previous method [25]. Mitochondrial NADH–ubiquinone reductase (complex I), succinate–CoQ oxidoreductase (complex II), CoQ–cytochrome c reductase (complex III), cytochrome c oxidase (complex IV), and ATP synthase (complex V) activities were detected as previously described [25].

### 2.6. Western Blot 

Liver and heart proteins were extracted and determined using a BCA Protein Assay kit (Pierce, Rockford, IL, USA) as previously described [25]. Then, 10–20 μg of the extracted protein samples were subjected to SDS-polyacrylamide gel electrophoresis (SDS-PAGE), transferred to nitrocellulose membranes, and blocked with 5% nonfat milk. Then the blocked membranes were incubated with primary antibodies overnight followed by horseradish peroxidase-conjugated secondary antibodies for 1 h, developed by using an ECL detection kit (Pierce, Rockford, IL, USA) and quantified by scanning densitometry. As loading controls, the expression of each protein was adjusted to that of β-actin for the liver or GAPDH for the heart.

### 2.7. Protein Carbonylation Assay

Protein carbonyls were analyzed by using an Oxyblot protein oxidation detection kit (Cell Biolabs, San Diego, CA, USA). Equal amounts of the protein samples were subjected to SDS-PAGE followed by staining with Coomassie brilliant blue as a loading control. 

### 2.8. Real-Time PCR

Liver and heart total RNA was extracted by using a TRIzol reagent (Invitrogen, Carlsbad, CA, USA), reverse-transcribed into cDNA by using an RT-PCR kit (TaKaRa, Dalian, China), and quantified by real-time PCR with the primers presented in Table S1. Total DNA was extracted by using a QIAamp DNA Mini Kit (Qiagen, Hilden, Germany) and quantified by real-time PCR with mitochondrial D-loop primers for mtDNA copy number analysis. The 2^−ΔΔCt^ method was adopted to analyze both the mRNA and DNA results, and 18S rRNA was used as a housekeeping gene.

### 2.9. Echocardiography

Cardiac ultrasound was determined by using a Visual Sonics Vevo 770 Imaging System. The diastolic interventricular septum thickness (IVSd), systolic interventricular septum thickness (IVSs), left ventricle (LV) diastolic internal diameter (LVIDd), LV systolic internal diameter (LVIDs), LV diastolic posterior wall thickness (LVPWd), and LV systolic posterior wall thickness (LVPWs) were recorded. LV mass, LV diastolic volume (LV-Vol-d), LV systolic volume (LV-Vol-s), LV ejection fraction (EF), and fractional shortening (FS) were calculated according to the standard formulae.

### 2.10. Statistical Analysis

One-way ANOVA was used to analyze the data in all the experiments, and *p* < 0.05 was regarded as statistically significant. Data are presented as the mean ± S.E.M.

## 3. Results

### 3.1. HAE Improved Hyperlipidemia and Ameliorated Hepatic Lipid Metabolic Disorders

**Figure 1:**
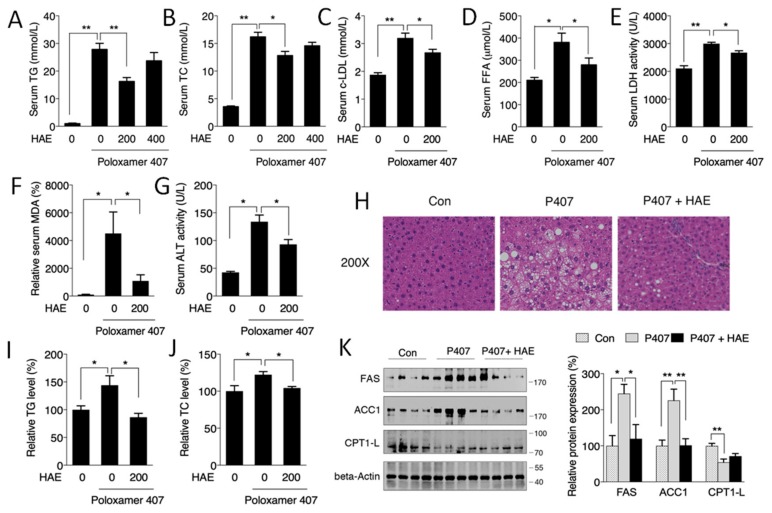
*Herba houttuyniae* aqueous extract (HAE) attenuated serum hyperlipidemia and ameliorated hepatic lipid metabolic disorders. (**A**) Serum TG content. (**B**) Serum TC level. (**C**) Serum c-LDL level. (**D**) Serum FFA level. (**E**) Serum LDH activity. (**F**) Serum MDA level. (**G**) Serum ALT activity. (**H**) H&E staining of liver tissue. (**I**) Liver TG level. (**J**) Liver TC level. (**K**) Liver FAS, ACC1, and CPT1L protein expression (left, western blot image; right, statistical analysis). The values are the means ± S.E.M. (*n* = 8); * *p* < 0.05; ** *p* < 0.01.

As shown in Figure 1A,B, P407 treatment markedly increased the serum TG and TC contents by more than 20-fold and 4-fold, respectively, indicating the successful establishment of a mouse model of hyperlipidemia. Treatment with 200 mg/kg/day HAE effectively reduced the serum TG and TC contents, while 400 mg/kg/day of HAE had no obvious effects on either TG or TC. Thus, we only chose 200 mg/kg/day HAE treatment in the subsequent experiments. The levels of serum c-LDL, FFAs, and MDA and the activity of serum LDH were also noticeably increased by P407, and the HAE treatment significantly lowered c-LDL, FFA, and MDA content as well as LDH activity (Figure 1C–F). 

Previous studies have indicated that hyperlipidemia may take part in the development of fatty liver disease [5]. As illustrated in Figure 1H, the P407 group exhibited obvious lipid deposition, and HAE treatment restored the liver morphology to that of the normal controls. Consistently, hepatic TG and TC levels as well as serum ALT activity exhibited similar results to those of H&E staining (Figure 1G,I,J). The levels of ACC1 and FAS, key regulators of fatty acid synthesis, were markedly increased in the P407 group and were significantly decreased by the HAE treatment. The expression of CPT1L, a fatty acid transporter, was decreased in the P407 group, but HAE treatment failed to restore its expression (Figure 1K).

### 3.2. HAE Improved Cardiac Remodeling

**Figure 2:**
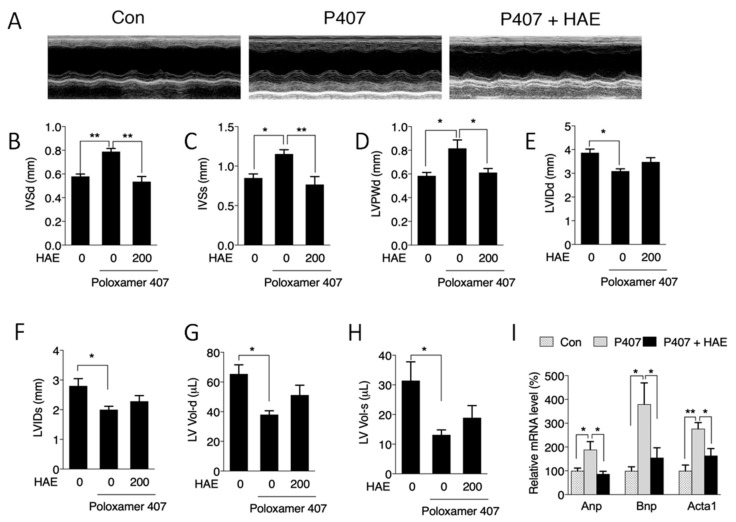
HAE improved cardiac remodeling. Con, control; P407, poloxamer 407; P407 + HAE, poloxamer 407 plus HAE at 200 mg/kg/day. (**A**) Echocardiogram images and quantitative analyses of (**B**) IVSd, (**C**) IVSs, (**D**) LVPWd, (**E**) LVIDd, (**F**) LVIDs, (**G**) LV-Vol-d, and (**H**) LV-Vol-s. (**I**) Heart mRNA contents of ANP, BNP, and ACTA1. The values are the means ± S.E.M. (*n* = 8); * *p *< 0.05; ** *p* < 0.01.

Evidence has indicated that the heart is another major organ damaged by excessive lipids [2,4]. Therefore, echocardiograms were recorded to detect the regulatory role of P407 and HAE in cardiac function. As shown in Figure 2A–H, P407 induced significant increases in IVSd, IVSs, and LVPWd and decreases in LVIDd, LVIDs, LV-Vol-d, and LV-Vol-s; and HAE treatment effectively decreased IVSd, IVSs, and LVPWd but failed to restore LVIDd, LVIDs, LV-Vol-d, and LV-Vol-s. However, neither P407 nor HAE had obvious effects on EF, FS, LVPWs, or LV mass (Figure S1). The mRNA levels of atrial natriuretic peptide (ANP), brain natriuretic peptide (BNP), and skeletal α-actin (ACTA1), key markers of cardiac hypertrophy and injury, were significantly upregulated in the P407 group and effectively reduced by HAE supplementation (Figure 2I). These results suggest that HAE is capable of improving cardiac remodeling induced by P407.

### 3.3. HAE Activated AMPK in Both the Liver and Heart

**Figure 3:**
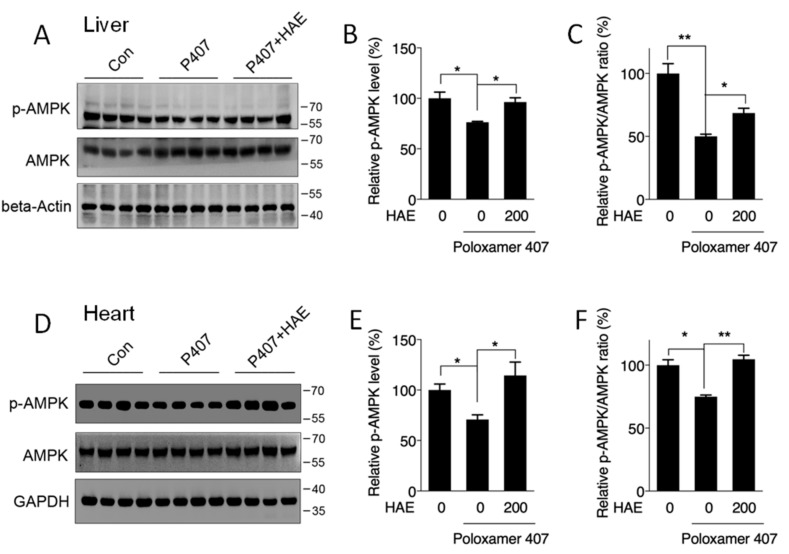
HAE activated the AMPK pathway in mice livers and hearts. Con, control; P407, poloxamer 407; P407 + HAE, poloxamer 407 plus HAE at 200 mg/kg/day. Total proteins were prepared from mice livers and hearts, and p-AMPK as well as AMPK protein contents were determined by western blot. (**A**) Western blot image, (**B**) the statistical analysis of p-AMPK, and (**C**) the ratio of p-AMPK/AMPK in mice livers. (**D**) Western blot image, (**E**) the statistical analysis of p-AMPK, and (**F**) the ratio of p-AMPK/AMPK in mice hearts. The values are the means ± S.E.M. (*n* = 8); * *p *< 0.05; ** *p* < 0.01.

We next determined the involvement of AMPK in hyperlipidemic mouse livers and hearts. Our results showed that P407 induced obvious declines in the p-AMPK level as well as in the p-AMPK/AMPK ratio in both the liver and heart (Figure 3A–F). HAE treatment significantly recovered both the p-AMPK level and the p-AMPK/AMPK ratio in the livers and hearts of hyperlipidemic mice (Figure 3A–F), suggesting a significant activation of AMPK by HAE.

### 3.4. HAE Attenuated Oxidative Stress by Activating the Phase II Enzyme Pathway 

**Figure 4:**
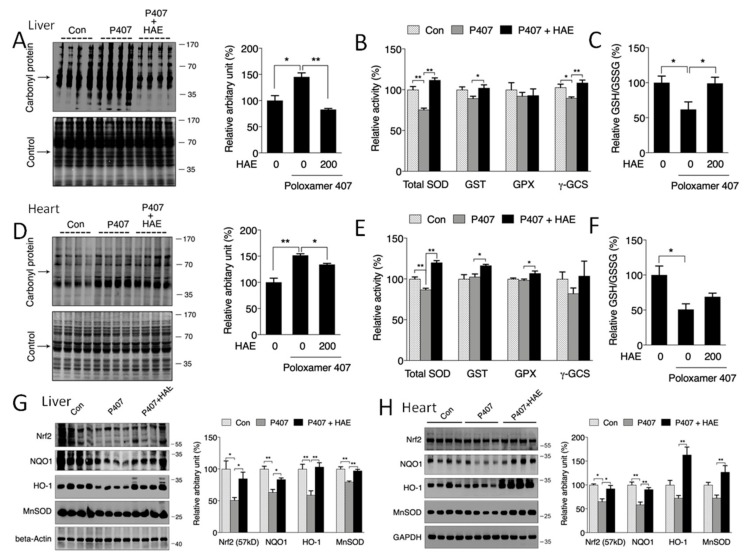
HAE ameliorated oxidative stress by upregulating the phase II enzyme pathway in mice livers and hearts. Con, control; P407, poloxamer 407; P407 + HAE, poloxamer 407 plus HAE at 200 mg/kg/day. (**A**) Liver protein carbonyl content (left, western blot image; right, statistical analysis). (**B**) Liver SOD, GST, GPX, and γ-GCS activities. (**C**) The liver GSH/GSSG ratio. (**D**) Heart protein carbonyl content (left, western blot image; right, statistical analysis). (**E**) Heart SOD, GST, GPX, and γ-GCS activities. (**F**) The heart GSH/GSSG ratio. (**G**) Liver Nrf2, NQO1, HO1, and MnSOD protein expression (left, western blot image; right, statistical analysis). (**H**) Heart Nrf2, NQO1, HO1, and MnSOD protein expression (left, western blot image; right, statistical analysis). The values are the means ± S.E.M. (*n* = 8); * *p* < 0.05; ** *p* < 0.01.

To determine whether HAE could improve hyperlipidemia-induced oxidative stress, a protein carbonyl assay was adopted to detect protein oxidative status. Our results showed that the carbonyl protein contents in both the livers and hearts of the P407 group were obviously increased compared to those of the control group (Figure 4A,D). HAE supplementation effectively inhibited the elevation of protein carbonyl levels in both the liver and heart. The phase II pathway is reported to play a vitally important role in fighting oxidative stress [34]. Our previous study elucidated that AMPK was able to regulate phase II enzyme expression [21,22]. Thus, we next evaluated the protein expression of phase II enzymes in the liver and heart. As shown in Figure 4G,H, P407 significantly decreased Nrf2, NQO1, HO-1, and MnSOD protein contents in the liver and decreased Nrf2 and NQO1 protein contents in the heart, whereas HAE treatment effectively increased all four protein levels in both the liver and heart compared to the P407 group (Figure 4G,H). P407 treatment also reduced total SOD and γ-GCS activity in the liver and total SOD activity in the heart, and HAE treatment sufficiently improved the total SOD and GST activity in both the liver and heart and increased γ-GCS activity in the liver as well as GPX activity in the heart (Figure 4B,E). Consistently, P407 obviously reduced the GSH/GSSG ratio in both the liver and heart, and HAE treatment markedly increased the GSH/GSSG ratio in the liver but failed to restore it in the heart (Figure 4C,F). Taken together, all of these results indicate that HAE can upregulate phase II enzymes and attenuate oxidative damage in both the livers and hearts of mice treated with P407.

### 3.5. HAE Promoted Mitochondrial Biogenesis and Mitochondrial Complex Activity

**Figure 5:**
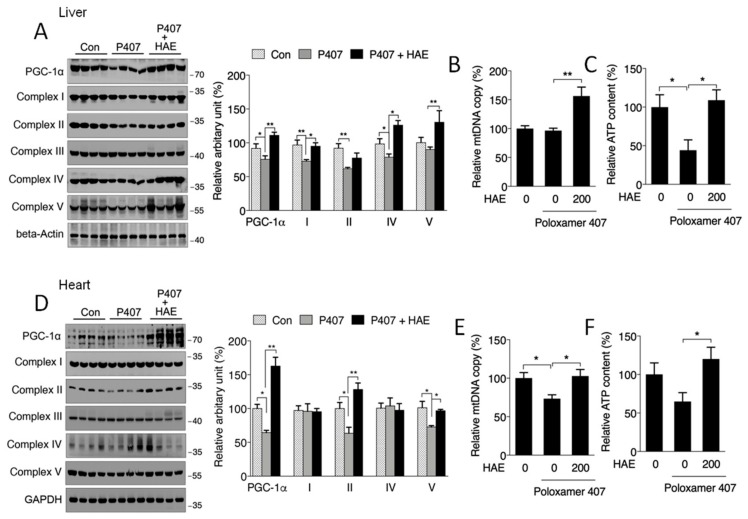
HAE activated the mitochondrial biogenesis pathway in mice livers and hearts. Con, control; P407, poloxamer 407; P407 + HAE, poloxamer 407 plus HAE at 200 mg/kg/day. (**A**) Protein levels of liver PGC-1α and mitochondrial complex subunits I–V (left, western blot image; right, statistical analysis). (**B**) Liver mtDNA copy number. (**C**) Liver ATP level. (**D**) Protein levels of heart PGC-1α and mitochondrial complex subunits I–V (left, western blot image; right, statistical analysis). (**E**) Heart mtDNA copy number. (**F**) Heart ATP level. The values are the means ± S.E.M. (*n* = 8); * *p* < 0.05; ** *p* < 0.01.

Mitochondria are vitally important organelles in regulating cellular metabolism. It has been reported that AMPK can promote mitochondrial biogenesis via the activation of PGC-1α [15,21,22]. Thus, we next investigated whether mitochondrial biogenesis is affected in the livers and hearts of hyperlipidemic mice. As shown in Figure 5A,D, we found the downregulated protein expression of PGC-1α and mitochondrial complexes I, II, and IV in the liver as well as of PGC-1α and mitochondrial complexes II and V in the heart of P407-treated mice, and the levels of most of these proteins were sufficiently improved by HAE treatment except for the level of complex II in the liver. Moreover, HAE treatment improved complex V activity in the liver, even though the reduction of complex V activity induced by P407 was not obvious (Figure 5A). We also found that P407 significantly reduced the mtDNA copy number in the heart and lowered ATP production in the liver. HAE treatment effectively increased both indicators in the liver and heart, even though the mtDNA copy number in the liver and ATP production in the heart were not obviously changed in the P407 group (Figure 5B,C,E,F). 

**Figure 6:**
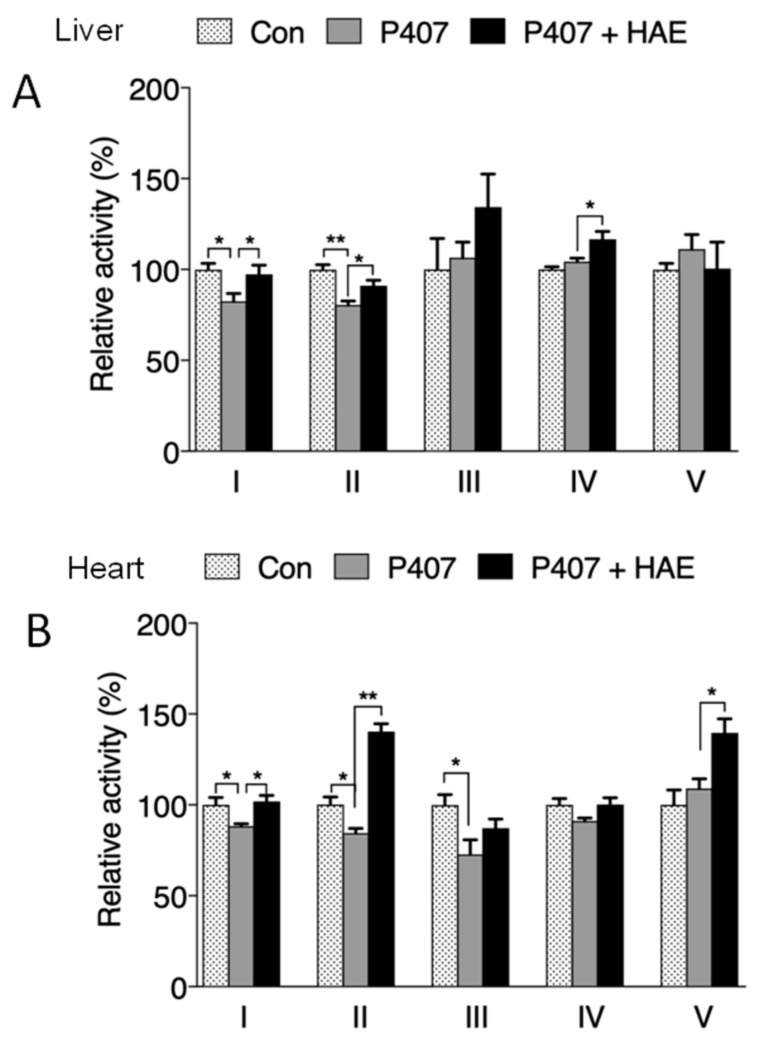
HAE improved mitochondrial complex activity in mice livers and hearts. Con, control; P407, poloxamer 407; P407 + HAE, poloxamer 407 plus HAE at 200 mg/kg/day. (**A**) Liver mitochondrial complex I–V activity. (**B**) Heart mitochondrial complex I–V activity. The values are the means ± S.E.M. (*n* = 8); * *p* < 0.05; ** *p* < 0.01.

The activities of mitochondrial complexes are strongly associated with mitochondrial oxygen consumption capacity and ATP production. As illustrated in Figure 6A,B, P407 treatment obviously reduced complex I and II activity in both the liver and heart, and HAE supplementation effectively prevented the reduction of activity in complex I and II in the liver and heart but failed to restore complex III activity in the heart. Although P407 did not show obvious effects on liver complex IV activity and heart complex V activity, HAE treatment significantly increased the activity of both complexes (Figure 6A,B).

## 4. Discussion

As one of the major risk factors of MS, hyperlipidemia is closely linked to the occurrence of fatty liver disease, cardiovascular disease, and many other metabolic diseases, of which the detailed mechanisms are still poorly understood [2,4,5,6]. A high-fat diet-induced model [22,25] and ApoE−/− transgenic mice are commonly used as animal models to investigate dyslipidemia-associated metabolic disorders. However, the direct role of hyperlipidemia in metabolic abnormalities and impairment in different tissues (except for blood vessels) is still largely unclear. P407 is a widely used nonionic surfactant that could cause hyperlipidemia and atherosclerosis in rodents [27]. In addition to vascular endothelial dysfunction, P407-induced models show abnormalities in tissues, including the liver [27], heart [35], and hippocampus [36], and a 0.5 g/kg P407 injection in mice for 24 h is enough to induce hyperlipidemia [37]. Therefore, we used one injection of 0.5 g/kg P407 to explore the underlying mechanism of acute hyperlipidemia-induced metabolic disorders and impairment in mice livers and hearts. 

Although many biological functions have been proposed for *Herba houttuyniae* [28,29,30,31,32], its effects on MS and hyperlipidemia-associated abnormalities remain largely uncharacterized. The main component of fresh *Herba houttuyniae* is water, and no major components account for large proportions of the dried weight of *Herba houttuyniae* [28]. A few isolated phytochemicals of *Herba houttuyniae*, including houttuynia sodium, flavonoids, and alkaloids, have been identified as having biological activity [38,39,40], while their presence in only small amounts in *Herba houttuyniae* restricts further exploration. Moreover, evidence has shown that the water extract of *Herba houttuyniae* exhibits superior biological activity [41]. Thus, the HAE used in our study was a water-extracted dried powder. We chose low (200 mg/kg/day) and high doses (400 mg/kg/day) of HAE based on previous animal studies [32,41] and found that both P407 and a low dose of HAE did not have obvious effects on body, liver, and heart weight. However, a high dose of HAE slightly decreased liver weight compared to the control group, indicating mild hepatic toxicity (Figure S2A–C). Interestingly, the injection of P407 successfully induced a model of hyperlipidemia with sharply increased serum TG and TC contents, and 200 mg/kg/day HAE significantly decreased the serum TG and TC contents, while 400 mg/kg/day HAE failed to do so (Figure 1A,B). Thus, we chose the 200 mg/kg/day dose of HAE for subsequent experiments.

Evidence has suggested that excessive FFAs can exaggerate ROS levels and induce oxidative stress [42]. Thus, we detected serum MDA content, a marker of oxidative damage to lipids, proteins, and DNA. Consistently, sharply elevated MDA levels induced by P407 were effectively alleviated by HAE treatment (Figure 1F). Serum LDH, ALT, and AST activities are commonly considered to be markers of tissue dysfunction, mainly for the heart and liver [43,44]. Our results showed that all of these enzymes were obviously increased by P407, indicating cardiac and hepatic impairments. HAE treatment lowered the activity of both LDH and ALT (Figure 1E,G). 

Next, we determined whether P407 and HAE could affect the morphology and function of mice livers and hearts. Our data showed obvious lipid deposition and lipogenic induction in mice livers after P407 injection, and all of these abnormalities were ameliorated by HAE supplementation (Figure 1H–K). Regarding cardiac remodeling, we found that P407 induced significant increases in cardiac IVSd, IVSs, and LVPWd and decreases in LVIDd, LVIDs, LV-Vol-d, and LV-Vol-s, indicating cardiac morphological changes caused by hyperlipidemia, and some of these changes were effectively attenuated by HAE treatment (Figure 2A–H). Moreover, HAE treatment significantly downregulated mRNA levels of cardiac hypertrophy and injury markers ANP, BNP, and ACTA1, which were all highly induced by P407 (Figure 2I). Interestingly, neither P407 nor HAE had obvious effects on cardiac EF and FS, suggesting that 24-h acute hyperlipidemia may not be enough to cause changes in cardiac function.

AMPK is a well-known energy sensor and metabolic regulator that promotes catabolic pathways such as fatty acid oxidation and inhibits anabolic pathways such as lipogenesis. Although recent investigation has suggested a possible link between hyperlipidemia and the AMPK pathway in the vascular system [23,24], the regulatory role of AMPK in hyperlipidemia-induced impairment in other tissues, except for the vascular system, remains largely unclear. In our current research, we discovered a deficiency in AMPK activity in both the liver and heart after P407 treatment, and HAE treatment restored AMPK activity in both of these tissues (Figure 3A–F). A previous study reported that AMPK can downregulate the lipogenic genes *ACC1* and *FAS* by mediating SREBP-1 and ChREBP [45]. Our results also showed decreased ACC1 and FAS protein contents in P407-treated mice livers, which may be attributable to the decreased hepatic AMPK activity. 

In addition to lipid metabolism, AMPK can also modulate oxidative stress as well as mitochondrial function by regulating the Nrf2-mediated phase II enzyme pathway and PGC-1α-mediated mitochondrial biogenesis, both of which exhibit a close correlation with hyperlipidemia-related metabolic disorders [8,10,11,12,46]. In our research, we observed that P407 markedly reduced the protein levels of Nrf2 and its downstream genes *NQO1*, *HO-1*, and *MnSOD* in the liver and *NQO1* in the heart, increased protein carbonyl levels in both the liver and heart, and reduced the ratio of GSH/GSSG in the heart, all of which were restored by HAE treatment (Figure 4A,C,D,F–H). The involvement of mitochondria in metabolic regulation is inevitable, but the specific role of mitochondria in hyperlipidemia-induced metabolic disorders still needs to be further elucidated. As expected, our results exhibited reduced protein levels of PGC-1α and mitochondrial complex subunits in both the liver and heart after P407 treatment. Meanwhile, a reduced mtDNA copy number in the heart and decreased ATP content in the liver were also observed after P407 injection, and HAE treatment not only upregulated mitochondrial biogenesis-related proteins but also increased the mtDNA copy number and enhanced ATP production in both the liver and heart (Figure 5A–F). Decreased mitochondrial biogenesis is always correlated with impaired mitochondrial oxidative capacity, which plays a key role in the pathogenesis of MS [12]. Consistently, P407 injection reduced mitochondrial complex activities in both the liver and heart, and most of the complex activities were restored by HAE treatment (Figure 6A,B). To further confirm the beneficial effects of HAE, we also adopted a palmitic acid (PA)-induced HepG2 cellular model with or without HAE pretreatment. Consistently, HAE significantly decreased TG content, increased the p-AMPK/AMPK ratio, and enhanced mitochondrial oxygen consumption capacity in HepG2 cells treated with PA (Figure S3A–C). Mitochondrial network dynamics are also important in maintaining normal mitochondrial function. We found that the expression of mitochondrial fusion-related protein Mfn1 was reduced in both the liver and heart and that the fission-related protein Drp1 was reduced in the liver: all of these abnormalities were restored by HAE supplementation (Figure S4A–E). Interestingly, we found PA treatment obviously increased mitochondrial fission compared to the control group. Pretreatment with HAE increased mitochondrial fusion and therefore partially recovered mitochondrial morphology (Figure S5A). However, the detailed mechanisms involved still require further investigation. 

## 5. Conclusions

In conclusion, our work provides the first evidence that AMPK-mediated mitochondrial biogenesis and the Nrf2 pathway are involved in acute hyperlipidemia-induced liver and heart impairments and shows that HAE exerts protective effects on hyperlipidemia-related metabolic disorders and tissue impairments by upregulating the AMPK/PGC-1α/Nrf2 cascade. Altogether, our findings indicate that *Herba houttuyniae* may be an attractive AMPK activator for the prevention and treatment of hyperlipidemia-related abnormalities and diseases. Further studies are required to investigate the major active components of *Herba houttuyniae* and to establish its clinical applications.

## Supplementary Materials

The following are available online at https://www.mdpi.com/2072-6643/12/1/164/s1, Figure S1: Quantitative analyses of mice LVPWs, LV mass, EF, and FS; Figure S2: Mice body, liver, and heart weights; Figure S3: Protective effects of HAE on PA-induced HepG2 cells; Figure S4: Mitochondrial dynamic-related protein expressions in the liver and heart; Figure S5: Mitochondrial morphology in PA-induced HepG2 cells with or without HAE pretreatment; Table S1: Primers.

## Abbreviations

ACC1, acetyl-coenzyme A carboxylase 1; ALT, alanine aminotransferase; AMPK, adenosine monophosphate (AMP)-activated protein kinase; ATP, adenosine triphosphate; CPT1L, carnitine palmitoyltransferase-1L; c-LDL, cholesterol low-density lipoprotein; Drp1, dynamin-related protein 1; EF, ejection fraction; FAS, fatty acid synthase; FFA, free fatty acid; FS, fractional shortening; GAPDH, glyceraldehyde-3-phosphate dehydrogenase; GSH, glutathione, reduced; GSSG, glutathione, oxidized; GPX, glutathione peroxidase; GST, glutathione S-transferase; HAE, *Herba houttuyniae* aqueous extract; HO-1, heme oxygenase-1; IVSd, diastolic interventricular septum thickness; IVSs, systolic interventricular septum thickness; LDH, lactate dehydrogenase; LV, left ventricle; LVIDd, LV diastolic internal diameter; LVIDs, LV systolic internal diameter; LVPWd, LV diastolic posterior wall thickness; LVPWs, LV systolic posterior wall thickness; LV-Vol-d, LV diastolic volume; LV-Vol-s, LV systolic volume; MDA, malondialdehyde; Mfn1, mitofusin-1; Mfn2, mitofusin-2; MnSOD, manganese-containing superoxide dismutase; MS, metabolic syndrome; mtDNA, mitochondrial DNA; NQO1, NAD(P)H/quinone oxidoreductase; Nrf2, NF-E2 related factor; OPA1, optic atrophy 1; PA, palmitic acid; PGC-1α, peroxisome proliferator-activated receptor gamma coactivator-1 alpha; P407, poloxamer 407; ROS, reactive oxygen species; TC, total cholesterol; TG, triglyceride; γ-GCS, γ-glutamylcysteine synthetase.
