# Supplementary material for: Herba houttuyniae Extract Benefits Hyperlipidemic Mice via Activation of the AMPK/PGC-1α/Nrf2 Cascade
Source: Nutrients. 2020 Jan 7;12(1):164. doi: 10.3390/nu12010164 (PMC7019422; doi:10.3390/nu12010164)
Supplement: Supplementary file 1 [file nutrients-12-00164-s001.pdf]

Supplementary Materials

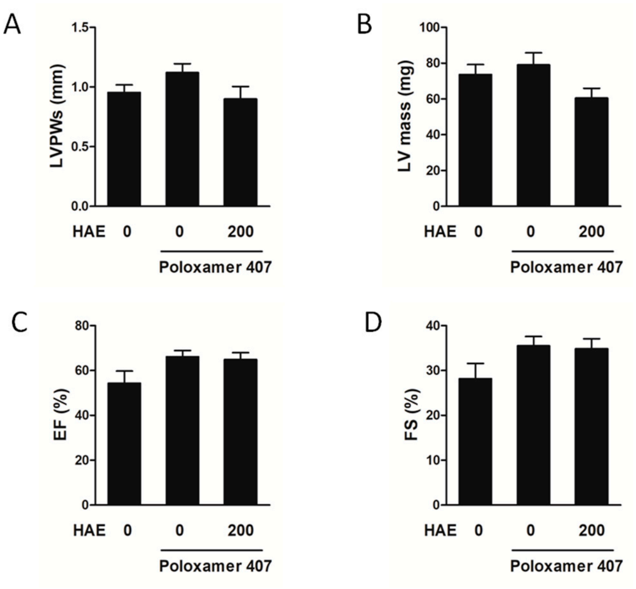

Figure S1. Quantitative analyses of mice LVPWs, LV mass, EF, and FS. (**A**) LVPWs. (**B**) LV mass. (**C**) EF. (**D**) FS. The values are the means ± S.E.M. (n = 8).

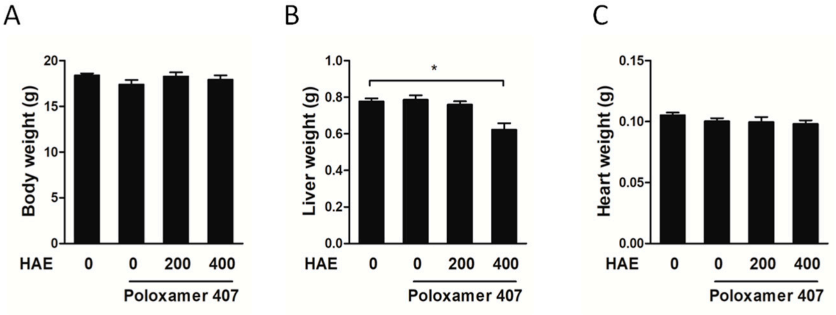

Figure S2. Mice body, liver, and heart weights. (**A**) Body weight. (**B**) Liver weight. (**C**) Heart weight. The values are the means ± S.E.M. (n = 8); * $p < 0.05$.

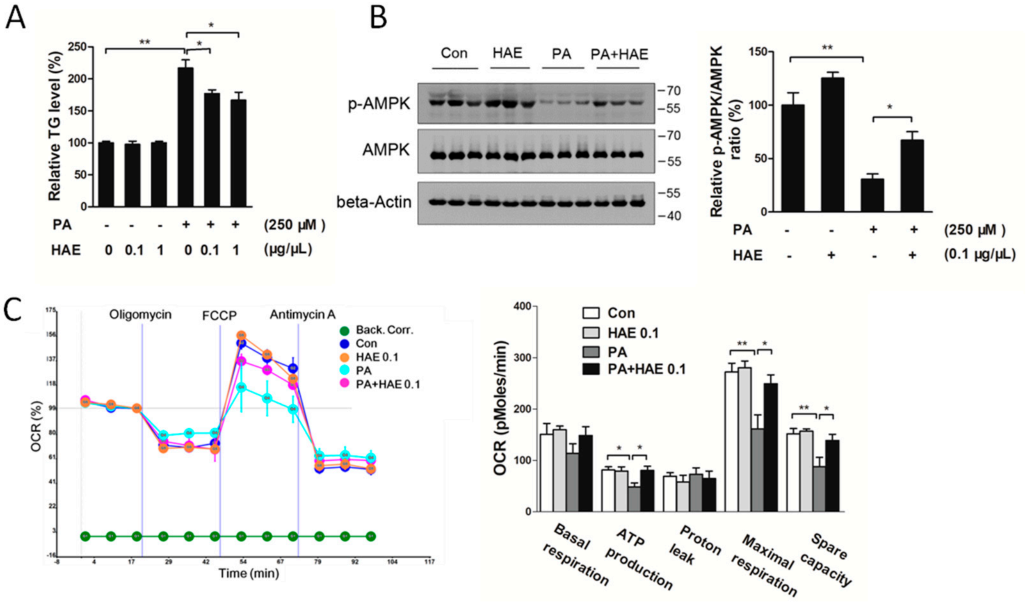

Figure S3. Protective effects of HAE on PA-induced HepG2 cells. HepG2 cells were pretreated with or without 0.1 μg/μL or 1 μg/μL of HAE for 24 h followed by 250 μM of PA challenge for another 24 h. Con, control; HAE, HAE at 0.1 μg/μL; PA, palmitic acid; PA + HAE, palmitic acid plus HAE at 0.1 μg/μL. (**A**) TG content. (**B**) p-AMPK and AMPK protein expression (left, western blot image; right, statistical analysis). (**C**) Mitochondrial oxygen consumption rate (OCR) (left, OCR image; right, statistical analysis). The values are the means ± S.E.M. (n = 3); * $p < 0.05$; ** $p < 0.01$.

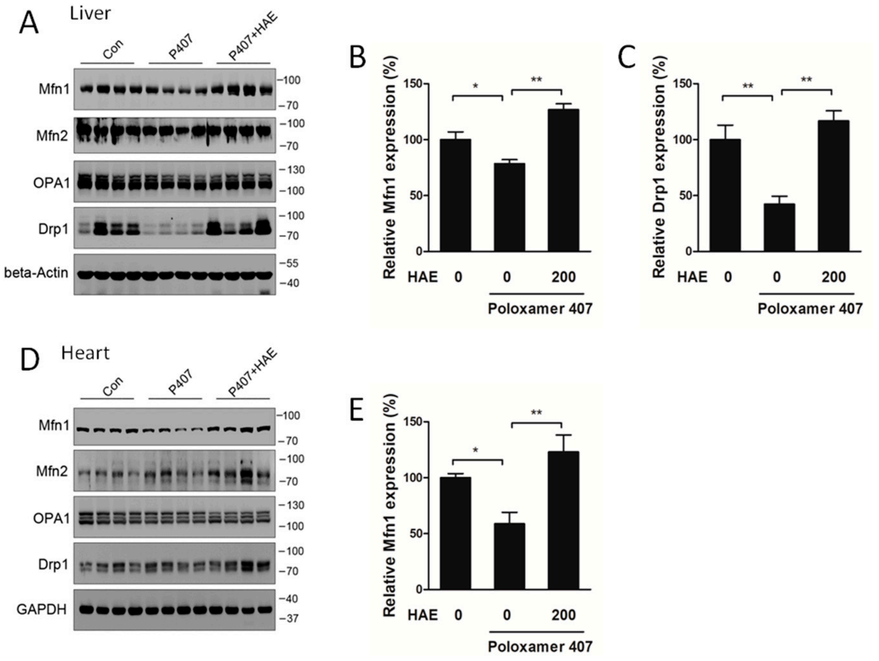

Figure S4. Mitochondrial dynamic-related protein expressions in the liver and heart. Con, control; P407, poloxamer 407; P407 + HAE, poloxamer 407 plus HAE at 200 mg/kg/day. The western blot image of (**A**) liver dynamic-related protein and (**D**) heart dynamic-related protein and the statistical analyses of (**B**) liver Mfn1 and (**C**) liver Drp1 contents and (**E**) heart Mfn1 content. The values are the means ± S.E.M. (n = 8); * $p < 0.05$; ** $p < 0.01$.

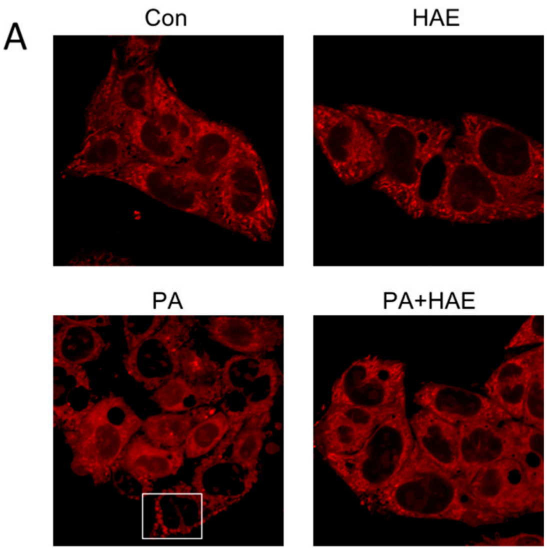

Figure S5. Mitochondrial morphology in palmitic acid (PA)-induced HepG2 cells with or without HAE pretreatment. HepG2 cells were pretreated with or without 0.1 μg/μL of HAE for 24 h followed by 250 μM of PA challenge for another 24 h. Then the cells were stained with Mito Tracker Red (Thermo) to mark mitochondrial morphology. (**A**) Confocal image of HepG2 cells. Con, control; HAE, HAE at 0.1 μg/μL; PA, palmitic acid; PA + HAE, palmitic acid plus HAE at 0.1 μg/μL.

Table S1. Primers.

Species	Gene	Primer (5’-3’)
Mouse	*18S rRNA*	Forward: GTAACCCGTTGAACCCCATT<br>Reverse: CCATCCAATCGGTAGTAGCG
Mouse	*ANP*	Forward: GATTTCAAGAACCTGCTAGACC<br>Reverse: AGTTTGCTTTTCAAGAGGGC
Mouse	*BNP*	Forward: CACCCAAAAAGAGTCCTTCG<br>Reverse: CAACAACTTCAGTGCGTTAC
Mouse	*ACTA1*	Forward: ACTGGGGACTAAATCCAAGTC<br>Reverse: CATACCTACCATGACACCCTGG
Mouse	*D-loop*<br>(for DNA)	Forward: AGGCATGAAAGGACAGCA<br>Reverse: TTGGCATTAAGAGGAGGG
Mouse	*18S rRNA*<br>(for DNA)	Forward: GAGAAACGGCTACCACATCC<br>Reverse: CACCAGACTTGCCCTCCA
